# Sindbis Virus Replication Reduces Dependence on Mitochondrial Metabolism During Infection

**DOI:** 10.3389/fcimb.2022.859814

**Published:** 2022-06-16

**Authors:** Juan L. Rodriguez, Jessica L. Costlow, Max Sheedy, Kelly T. Yoon, Annette M. Gabaldón, J. Jordan Steel

**Affiliations:** ^1^ Biology Department, Colorado State University- Pueblo, Pueblo, CO, United States; ^2^ Department of Biology, US Air Force Academy, Colorado Springs, CO, United States

**Keywords:** sindbis virus, mitochondria, metabolism, host cell manipulation, arbovirus

## Abstract

Alphaviruses are single stranded, positive sense RNA viruses that are often transmitted through mosquito vectors. With the increasing spread of mosquito populations throughout the world, these arboviruses represent a significant global health concern. Viruses such as Sindbis Virus (SINV), Chikungunya Virus (CHIKV) and Equine Encephalitis Viruses (EEV) are all alphaviruses. As viruses, these pathogens are dependent on the host cell environment for successful viral replication. It has been observed that viruses manipulate cellular metabolism and mitochondrial shape, activity, and dynamics to favor viral infection. This report looked to understand the metabolic changes present during Sindbis virus infection of hamster and human kidney cells. Cells were infected with increasing levels of SINV and at 24 hours post infection the mitochondria morphology was assessed with staining and mitochondrial activity was measured with a real-time Seahorse Bioanalyzer. The relative amount of mitochondrial staining intensity decreased with Sindbis virus infected cells. Both oxygen consumption rate and ATP production were decreased during SINV infection while non-mitochondrial respiration and extracellular acidification rate increased during infection. Collectively, the data indicates that SINV primarily utilizes non-mitochondrial metabolism to support viral infection within the first 24 hours. This understanding of viral preference for host cell metabolism may provide critical targets for antiviral therapies and help further define the nature of alphavirus infection.

## Introduction

Alphaviruses are single stranded, positive sense RNA viruses. The genus alphavirus includes Sindbis Virus (SINV), Chikungunya Virus (CHIKV), Equine Encephalitis Viruses (EEV), and a variety of mostly-arthropod transmitted viruses (Atkins, 2013). These viruses modulate the host cell environment to favor viral replication (Sanchez and Lagunoff, 2015; Fritsch and Weichhart, 2016). It has been shown that alphaviruses induce membrane changes, alter rates of glycolysis, and even affect autophagy/apoptosis. It is very common for +ssRNA viruses including SARSCoV2, Dengue viruses, and Alphaviruses to manipulate the host cell mitochondria to gain control of the infected cell and create a stable environment for viral replication (Gatti et al., 2020). Dengue virus, a mosquito borne flavivirus, upregulates mitochondrial activity including elongation of the mitochondria during infection (El-Bacha et al., 2007; Yu et al., 2015). This elongation aids the virus in reducing the free space pre-viral proteins need to travel through the cell in order to replicate more effectively (Barbier et al., 2016; Pagliuso et al., 2017). Venezuelen equine encephalitis virus, an alphavirus, has been shown to alter mitochondrial dynamics which have critical effects on viral replication (Keck et al., 2017). The mitochondria play critical roles in cell metabolism, antiviral activity, cell cycle regulation, and control of apoptosis/autophagy (Addabbo et al., 2009; Fujimoto and Hayashi, 2011; Khan et al., 2015). A better understanding of how alphaviruses modulate the host cell metabolism and particularly the mitochondria would lead to increased knowledge on how these global pathogens replicate.

There are limited options for treating alphaviruses. There is some published data showing that metabolic inhibitors such as imatinib mesylate can be used to reduce viral replication (Frantz and Wipf, 2010; Costlow et al., 2017). By further understanding alphavirus manipulation of the host cell metabolism, novel antiviral drug targets may be discovered that can be used to stop or reduce many pathogenic +ssRNA viral infections.

Sindbis virus is often used as the prototype alphavirus (Hernandez et al., 2010). This work looked at mitochondrial changes during Sindbis virus infection. Mitochondrial abundance, size, and activity were assessed to look for infections impact on the host cell mitochondria. It has been shown in published work that Sindbis virus lowers mitochondrial activity in infected neurons, but alphavirus have a wide cellular tropism and can infect kidney and liver cells (Silva da Costa et al., 2012; Findlay and Ulaeto, 2015). It is important to know how Sindbis virus affects various cell types and impedes metabolic and mitochondrial dynamics. The data presented here confirms that Sindbis virus reduces mitochondrial activity in both kidney and liver cells.

## Materials and Methods

### Cell Culture and Biological Material

Baby Hamster Kidney (BHK) (CCL-10) and Human Embryonic Kidney (HEK) (CRL-1573) were obtained from ATCC. Human hepatoma liver cells were obtained from Rushika Perera, who got them from the JCRB Cell Bank (JCRB0403 Huh-7). All cells were grown in Dulbecco’s Modified Eagle Medium (DMEM) supplemented with 10% fetal bovine serum (FBS), 5% of a mix of Penicillin (10,000 units/mL), Streptomycin (10,000µg/mL), and Amphotericin (25 µg/mL) and 1% Ciprofloxacin (10 µg/mL). The cells were incubated at 37°C and 5% CO_2_ in 25 cm^3^ cell culture flasks and split at 85% confluency (every 2-3 days) to maintain metabolic rates analyzed in experiments.

### Viral Infection

Cells were passaged, counted, and plated into 12, 24, or 48 well plates for the experiment. The default concentration of cells was 75,000 cells/mL. After 24 hours of the cells being attached and growing, a sample set of wells were counted using trypsinization and trypan blue quantification with a hemocytometer to determine the number of cells in each well. Virus was then added to the corresponding wells at the calculated multiplicity of infection (MOI, 0.1, 1, and 10). Virus stocks of Sindbis virus (AR339) and dsSINV were maintained at -80C and the stock was diluted using cell culture media. The diluted virus media at the specified concentration was then added to the cells at time 0 of infection. The infection proceeded for the allotted time until the treatment or experimental analysis was performed. Plaque assays were routinely performed to confirm the presence of infectious virus in viral stocks and samples. The reporter virus has been shown to accurately reflect infectious plaque forming units (Steel et al., 2011). 24 hours post infection was selected as a standard time for assays because that allows sufficient time for the viral infection to take place, while ensuring high cell viability (data not shown).

### Mitochondrial Staining and Flow Cytometry

To determine if there were any effects on the host cell mitochondria during Sindbis Virus (SINV) infection, the average density of Mitotracker fluorescence was measured *via* flow cytometry. BHK and HEK cells were cultured and allowed to grow for 24 hours. Those cells were then infected with Sindbis virus at various multiplicities of infection (MOIs) for 24 additional hours, and then incubated in a solution of 100uM Mitotracker for approximately 30 minutes before being run through the flow cytometer.

Cells were plated in 24-well plates at 75,000 cells per mL and allowed to grow for 24 hours. The old media was then removed, and the cells were incubated in a solution of PBS and 100 µM green mitotracker dye (Biotium, MitoView Green) for 30 minutes. The cells were rinsed with PBS, trypsinized, and diluted with media, then transferred to 1.5 mL microfuge tubes. The cells were spun down at 2,000 xG for one minute at room temperature, the media was replaced with PBS, then run through the flow cytometer and the average green fluorescence per cell was analyzed. The Millipore Guavasoft flow cytometer was used for all experiments.

### Drug Treatment

Kidney cells were plated at 75,000 cells per mL, in a 48 well plate with 250 µL of cell suspension in each well. Serial dilutions of MFP (mitochondrial fusion promoter) were made to test its efficiency as an antiviral treatment. The highest concentration used was 100 µM, and each concentration following was half of its previous. Cells were infected with dsSINV expressing mCherry (Steel et al., 2011) at various MOI’s/concentrations, and the red fluorescence levels were detected on the flow cytometer at a wavelength of 561 nm at each concentration of the drug used. The percentage of highly infected cells were quantified and graphed to show comparison from one concentration of drug to the next.

### Lactate Assay

Lactate accumulation over a 24 hour-period was measured in uninfected cells (MOCK) and Sindbis infected cells (SINV) using an enzymatic lactic acid assay kit (Sigma-Aldrich, MAK064). Solutions were prepared per protocol requirements and used for the following experimentation. Huh7 cells were passaged and plated at 6X10^4^ cells per well in a 24 well plate (Celltreat, 229124) as in previous experiments and incubated as usual for 24 hours. Cells were infected with a MOI of 1 and incubated for one hour, followed by a change in media. An MOI of 1 was used to induce significant infection level (see **Supplementary Figure 1**). After 24 hours post infection, supernatants from both MOCK and SINV were saved for analysis, as described below. In preparation of extracellular lactate, the supernatant samples were first spun at 500 rpm for 3 minutes to remove any suspended HEK cells that may have detached during incubation. The samples were then centrifuged at 13,000XG for 10 minutes at 4°C in a 10 kDa molecular weight cut-off spin filter (Sigma Aldrich, GE28-9322-47) to remove any lactate dehydrogenase (LDH) that may consume lactate and were stored at -80°C until subsequent analysis. Samples were analyzed using a 96 well plate (Celltreat, 229196), and data were recorded using a colorimetric plate reader at 570 nm. Samples were prepared for analysis by adding 2 μL of each sample into its respective well containing 98 μL of lactate solution and incubated before analysis. A lactate standard was generated for each analysis and used to derive the concentrations of lactate.

### Real-Time Metabolic Analysis

Huh7 cells were passaged and plated as previously described with fresh 1X DMEM into a XFe24 plate (Agilent Technologies, 102340-100). Huh7 cells were incubated for one hour in a humidified 5% CO_2_ infused incubator and cells were further processed through standard protocol and incubated for 24 hours before infection.

The day prior to the assay, after the 24-hour cell incubation period, cells were treated as described above. Further perpetration for the real-time metabolic analysis was needed and was done by placing 1 mL of calibrant solution (Agilent Technologies, 102340-100) into the Seahorse sensor cartridge with drug ports (Agilent Technologies, 102340-100) and was placed into a 37°C non-CO_2_ incubator. Also, 48.8 mL of non-supplemented Seahorse XF base medium (Agilent Technologies, 102353-100) was placed into a 37°C non-CO_2_ incubator for 1 hour.

On the day of the assay, the 48.8 mL of Seahorse XF base medium was supplemented with 1 mM pyruvate by adding in 500 μL of 100 mM pyruvate (Sigma-Aldrich, S8636), 2 mM L-glutamine by adding in 500 μL of 200 mM L-glutamine (Sigma-Aldrich, G8540), and 10 mM glucose by adding in 200 μL of 2.5 M glucose (Sigma-Aldrich, G8769) and pH to 7.4 with 0.1 M NaOH as per protocol for the Agilent mitostress test assay. This supplemented media was referred to as MST media.

Treated cells were processed by removing old media and washed two times with prepared MST media as per protocol recommendations. Cells then incubated in a non-CO_2_ incubator for 1 hour. During the one-hour incubation period, the mitostress test drug compounds were prepared using the MST media per manufacturer protocol. The drug compounds at their given dry weight were 63 nmol of oligomycin, 72 nmol of FCCP, and 27 nmol of both rotenone/antimycin a. The drug compounds were combined with MST to make the following stock concentrations: 100 μM for oligo, FCCP and 50 μM for R/A. Oligomycin is an inhibitor of complex V of the electron transport chain and inhibits the synthesis of ATP, which decreases the oxygen consumption rate (OCR). FCCP uncouples oxygen consumption from ATP synthesis and increases OCR. Rotenone and antimycin-a inhibit complex I and III, respectively. R/A together inhibit the mitochondrion completely shutting down the ETC, in turn decreasing OCR. The working solution for these compounds were then made by adding additional MST media. The working solution for each MST drug compound were loaded into their respective drug ports. The port concentrations start at 10 μM for both oligo and FCCP, and 5 μM for R/A. The final well concentration after injections would be 1.0 μM for both oligo and FCCP, and 0.5 μM for R/A.

Before analysis of the cells, the cartridge plate with calibrant solution was placed into the Seahorse. After the calibrant stage and the cells incubated in a non-CO_2_ incubator for one-hour, were analyzed on the Seahorse using the pre-set MST software protocol. After the metabolic analysis, cells were immediately trypsinized and a cell count was done for each well to determine the OCR measurements per total cells within each well. The OCR readings were normalized to the number of total cells within each well, using the control wells as the reference for each experiment.

### Statistical Analysis

One way ANOVAs, T-test, and Tukey post-hoc comparison tests were performed to determine significance in data and results. All statistical analysis was done using the software R. Values shown in figures are the mean and the error bars represent the Standard error of the mean, unless otherwise noted. P < 0.001 is indicated by ***. P<0.005 is indicated by *.

## Results

### SINV Infection Caused Decrease in Fluorescence Intensity of Mitochondria

**Figure 1:**
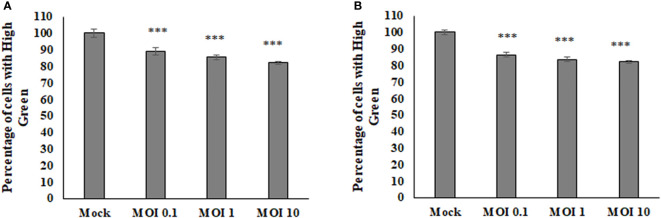
The average amount of fluorescence being picked up from the mitoView mitotracker decreases with increasing MOI in BHK cells **(A)**, HEK cells **(B)**.Statistical analysis was performed *via* a one way ANOVA and a tukey comparison test, where P < 0.001 is indicated by ***.

To determine if there were any effects on the host cell mitochondria during Sindbis Virus (SINV) infection, the average density of a mitochondrial fluorescence dye was measured *via* flow cytometry to assess relative mitochondria mass or abundance. There was a decrease in the fluorescence given off by the mitochondria with higher levels of infection in both HEK and BHK cell lines (**Figure 1**). These results indicate that the virus is manipulating the mitochondria during infection and perhaps lowering the abundance or staining of the mitochondria during infection.

### SINV Infection Causes Decrease in Oxygen Consumption and pH

**Figure 2:**
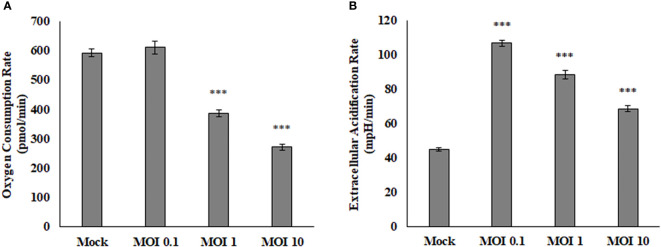
Oxygen consumption rate (OCR, **A**) is decreased during viral infection, while extracellular acidification rate (ECAR, **B**) is increased during viral infection. These HEK cells were infected at various MOIs and allowed to infect for 24 hours before reading results. These values are normalized to the baseline reading just before injection of Oligomycin. ***P < 0.005.

The Seahorse Bioanalyzer was used to determine respiration levels of the HEK cells during infection of various MOIs. This powerful instrument measures many variables that allow us to interpret where the energy within the cells is coming from and rule out pathways that the virus may or may not be using. Results showed a decrease in oxygen consumption rate (OCR) and an increase in extracellular acidification rate (ECAR) (**Figure 2**). The OCR is believed to be associated with the mitochondrial electron transport chain activity. If the rate of consumption is lowered, then it may be assumed that the activity of the electron transport chain is also lowered and the correlation continues if the values are increased. The ECAR is believed to be associated directly with lactate production and the rate of glycolysis. If the rate is lowered, then the amount of lactate being produced by the cells and the rate of glycolysis is also lowered and same if the values are increased. This data indicates that SINV is causing decreases in electron transport chain activity and higher levels of lactate production. Interestingly the ECAR was highest at low levels of infection, but as the infection level increased to an MOI of 10 the ECAR levels were lower than an MOI of 0.1. This may be due to other stress responses and cytotoxicity that the cells could be experiencing at a high level of infection at an MOI of 10. This also confirms that the infection is not killing the cells and causing acidification at this time post infection.

### SINV Infection Reduces ATP Production

**Figure 3:**
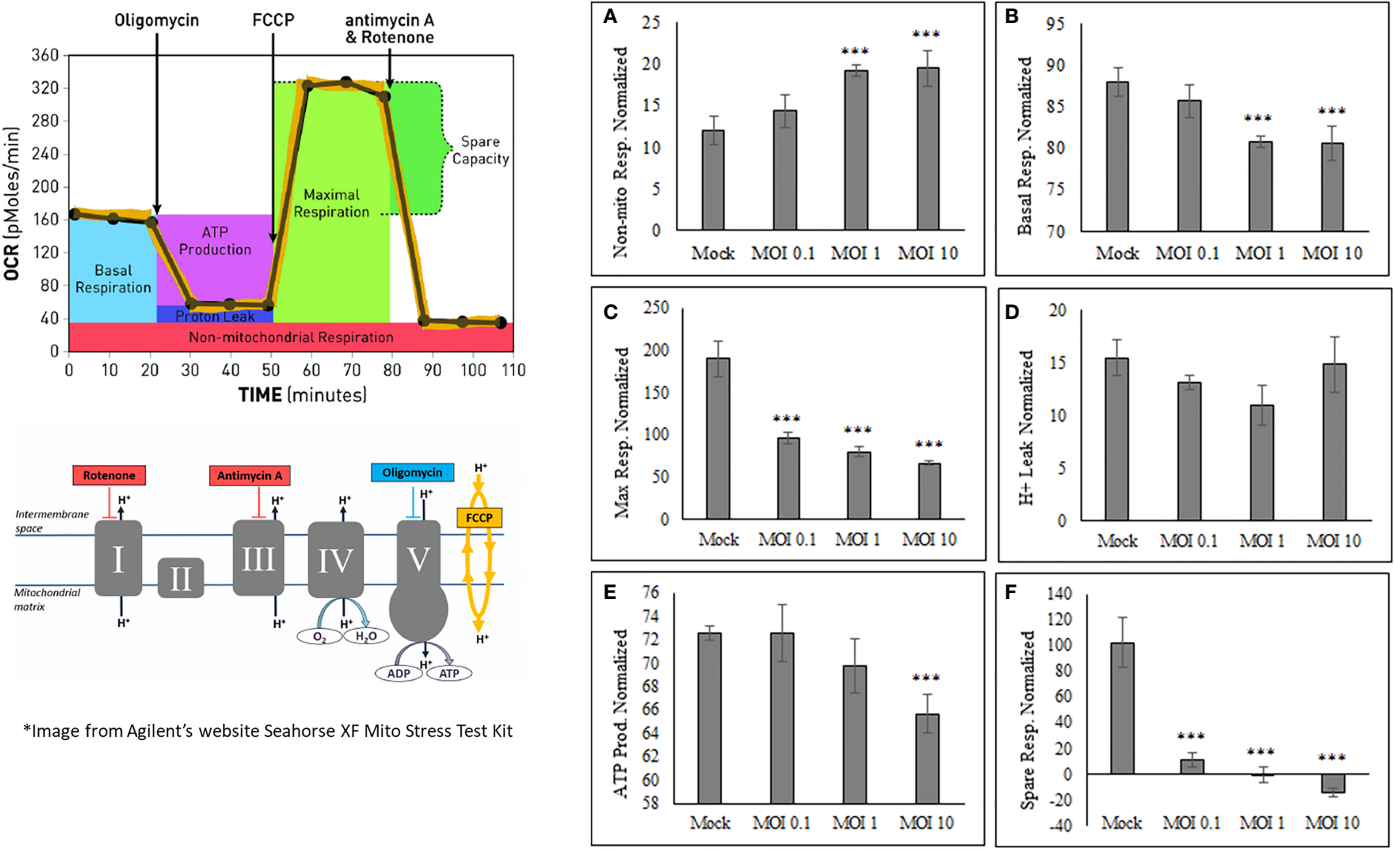
SINV causes decrease in ATP production and increase in non-mitochondrial respiration. The Seahorse data obtained shows an increase in only nonmitochondrial respiration **(A)**, with a decrease in basal respiration **(B)**, maximum respiration **(C)**, ATP production **(E)** and spare respiratory capacity **(F)**. No change was seen in proton leak **(D)**. These values are calculated from various time points within the OCR and ECAR readings using HEK cells. (***) indicates a p-value of less than 0.001.

After determining that oxygen consumption are affected during viral infection, it was important to determine what other mitochondrial characteristics were altered The OCR and ECAR values obtained from HEK cells using the Seahorse Bioanalyzer were used in calculating the basal respiration, maximal respiration, non-mitochondrial respiration, proton leak, ATP production, and spare respiratory capacity (**Figure 3**). Basal respiration is the baseline for normal oxygen consumption, whereas maximal respiration is the highest usage of oxygen possible, based on the uncoupling of ATP synthesis with FCCP treatment. Non-mitochondrial respiration is the oxygen consumed when the mitochondrial electron transport chain is completely inhibited by Rotenone treatment. The proton leak value is determined as the difference from when the ATP synthase enzyme is shut off (Oligomycin) and when the complete electron transport is shut off (Rotenone). ATP production is assessed based on the difference between basal respiration and inhibiting ATP synthesis (oligomycin). The spare respiratory capacity is the increase in Oxygen consumption when FCCP is used to uncouple the membrane and shows the difference between basal respiration and maximal respiration (**Figure 3**).

SINV infection of HEK cells increases Non-mitochondrial respiration (**Figure 3A**) while, Basal respiration, Maximum respiration, ATP production, and Spare respiratory capacity (**Figure 3** and **Supplementary Figure 2**) were all decreased as a result of active SINV infection. This data indicates that SINV is preferentially shuttling pyruvate into lactate production rather than into the Citric acid (TCA) cycle for maximal respiration. The decrease in basal respiration indicates that the electron transport chain is depressed during SINV infection. Overall, this data strongly indicates that SINV reduces host cell mitochondria activity during infection.

### Pyruvate to Acetyl-coA Shuttling

**Figure 4:**
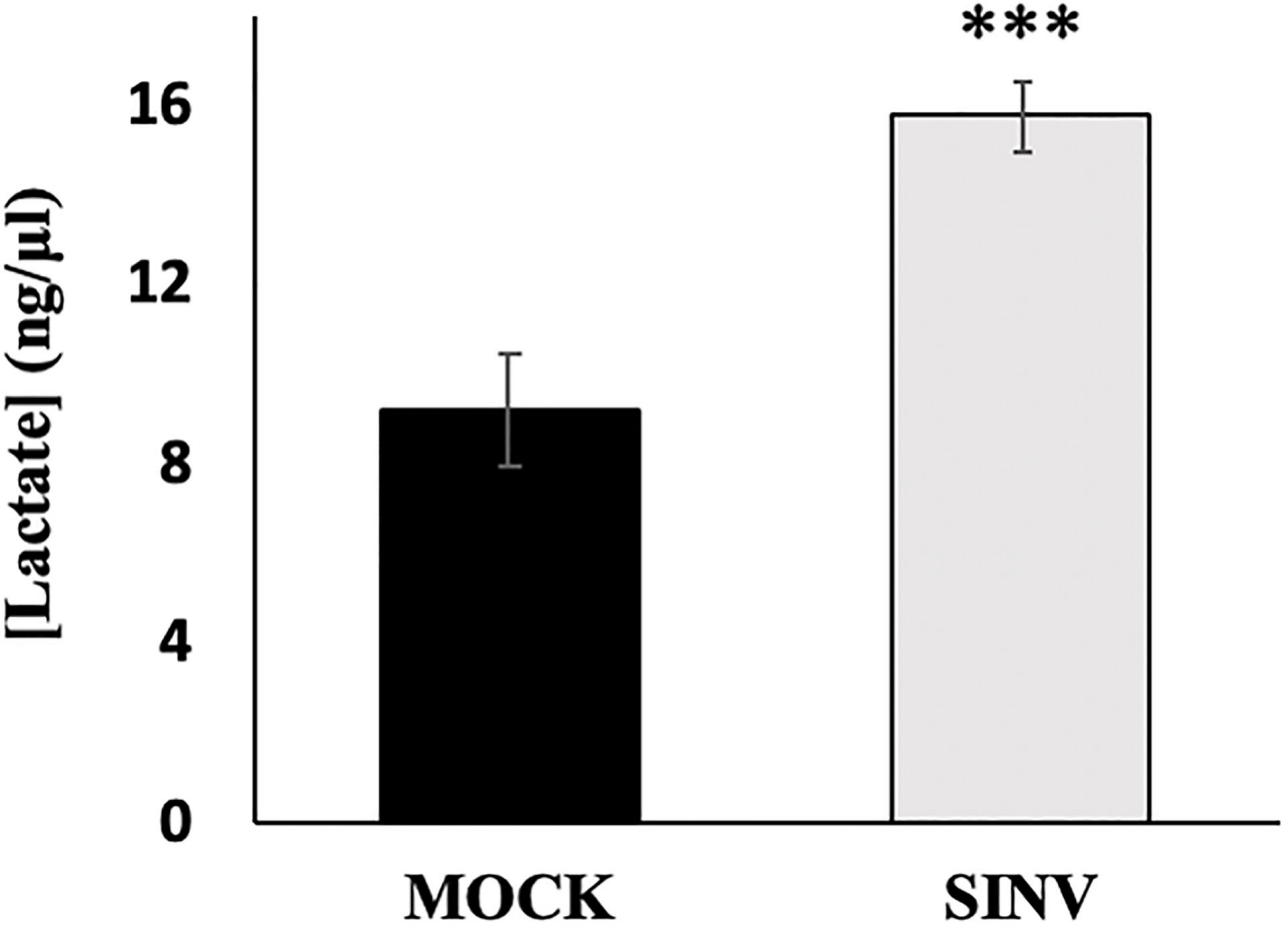
Lactic acid accumulation over 24 hours post infection. HEK cells infected with Sindbis virus (SINV) at an MOI=1 had significantly higher accumulations of lactate over 24 hours post infection. (***) P-value <0.001. Sample size n = 12.

Lactate is the final product through glycolysis during anaerobic respiration that can be used as an electron acceptor instead of oxygen. Previous studies have shown an extracellular accumulation in lactate after 24 hours of SINV infection, with the conclusion that glycolysis is elevated during infection (Gupta et al., 1988; Sirikutt and Kalayanarooj, 2014). Similarly, our results from a lactate acid accumulation assay show that SINV infected HEK cells (MOI=1) accumulate lactate as expected in comparison to other studies performed. Lactate accumulated more significantly over a 24-hour period in infected cells (**Figure 4**). Results were as follows, comparing Mock cells to infected untreated cells (9.18+1.24 vs. 15.78±0.78 ng/uL, p-value<0.001).

### Mitochondrial Stress Test During SINV Infection

**Figure 5:**
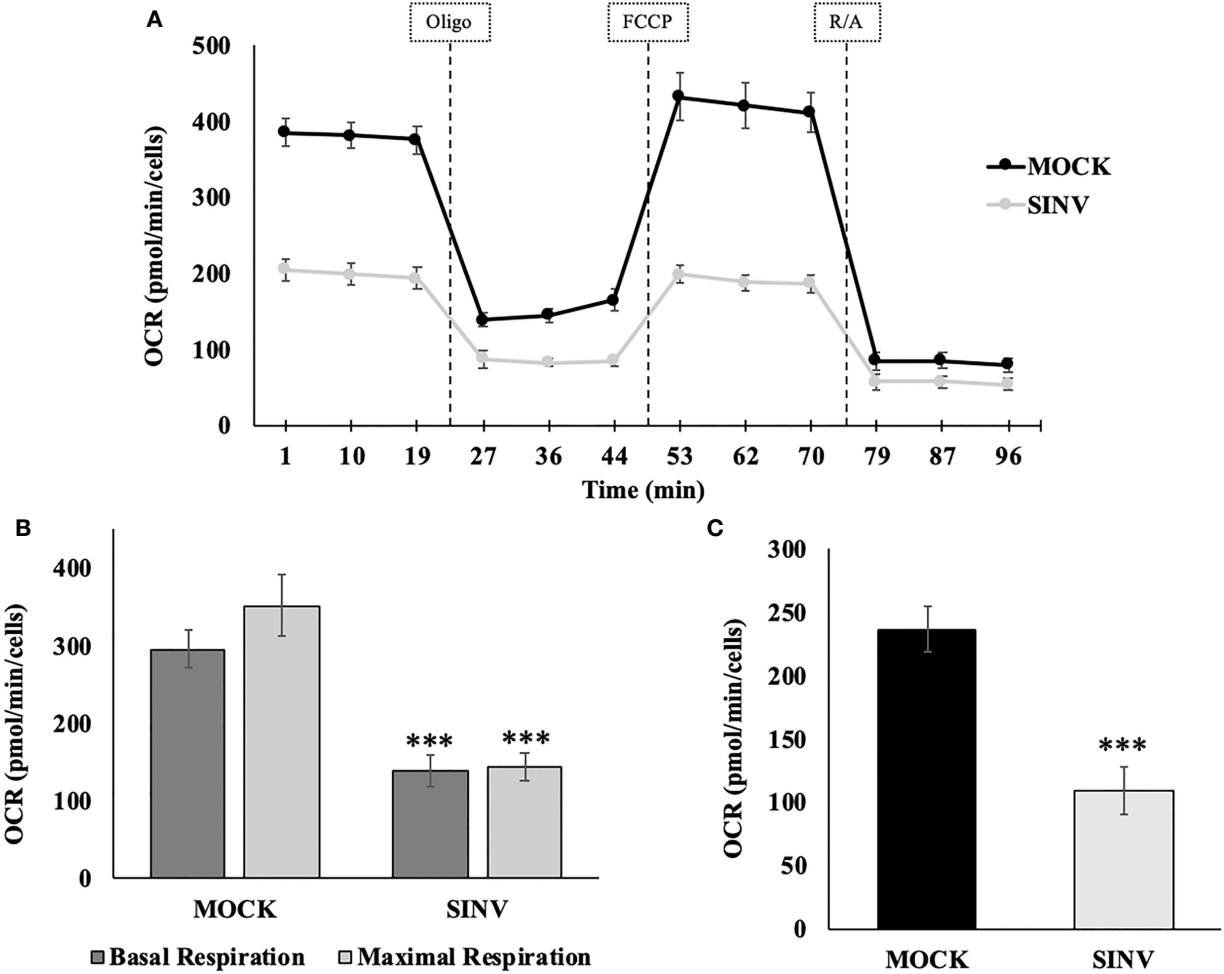
Reduced oxygen consumption at 24 hours post infection. Infected Huh7 cells experience a state of mitochondrial dysfunction with reduced OCR for the purpose of ATP synthesis. **(A)** Metabolic mitochondrial profiles during addition of mitochondrial inhibitors. **(B)** Basal and maximal respiration as determined by the oxygen consumption rate (OCR). **(C)** ATP production differences between uninfected (mock) and SINV infected (SINV). (***) P-value < 0.001. Sample size n = 9.

Mitochondrial respiration was evaluated using a mito stress test and an additional cell line, human hepatoma, Huh7 cells (**Figure 5**) for confirmation of the metabolic alterations during infection. Alphaviruses can also infect liver cells and we wanted to see if liver cells have similar mitochondrial dynamics as the infected kidney cells (HEK). The mito stress test compares the change in oxygen consumption rates (OCR) between uninfected cells (Mock) and infected Huh7 cells with Sindbis virus (SINV) by using various drug compounds that target the mitochondria’s electron transport chain (ETC) or its membrane. The first drug compound injected was oligomycin (oligo) which targets complex-V of the ETC and reduced OCR, the second drug compound was FCCP which uncouples oxygen utilization for ATP synthesis and increased OCR, and the final injection included the drug compounds rotenone/antimycin-a (R/A) which inhibited complex I and III, respectively, shutting down mitochondrial respiration and reduced (OCR). Cells infected with Sindbis failed to respire at normal levels compared to Mock cells. **Figure 5B** demonstrates oxygen utilization at basal and maximal conditions that would sustain energy consuming pathways. Basal respiration is the normal level of oxygen consumption to maintain cellular function and was derived from **Figure 5A** before the injection of oligo. Maximum respiration is the highest potential of the mitochondria to use oxygen uncoupled to ATP synthesis and was derived from **Figure 5A** after the addition of FCCP. Infected cells had a significantly lower OCR utilization for both basal and maximal respiration. The level of oxygen utilization for the purpose of ATP synthesis and was derived from **Figure 5A** after the addition of oligo and shows a significant drop in oxygen consumption for ATP synthesis in SINV infected cells (**Figure 5C**).

In order to determine the maximal capacity for respiration, FCCP was injected to uncouple mitochondrial respiration. Mock cells showed the highest respiratory capacity, with uncoupled OCR values increasing above basal OCR values. However, this was not the case for the SINV infected groups. With SINV infection, FCCP- induced response was no greater than the recorded basal OCR values. Infected cells appear to have little/no capacity for uncoupled respiration. With the final injection of rotenone/antimycin-a to shut down electron transport completely, OCR in both groups dramatically plummeted as expected. The final injection of R/A is to eliminate background noise related to other cellular processes that utilize oxygen other than mitochondrial respiration.

The results shown in **Figure 5** can be broken down further to understand several vital features of the mitochondria, such as basal respiration, ATP production rate, and maximal respiration. These results are summarized in **Figures 5B**, **C** for statistical comparisons between groups. Infected cells experienced a form of mitochondrial dysfunction that would lead to a decrease in ATP production. Basal OCR was significantly higher in Mock cells versus infected cells (OCR 296 ± 24 vs. 139 ± 22, p-value<0.001), **Figure 5B**. Maximal respiration was highest in Mock cells compared to infected cells. Mock cells significantly were able to increase OCR above basal values in response to FCCP, whereas infected groups barely met basal OCR levels or in some cases showed a reduced OCR. Results were as follows, comparing Mock cells to infected untreated cells (OCR 352 ± 39 vs. 145 ± 18, p-value<0.001), **Figure 5B**.

Synthesis of ATP is dependent on H^+^ pumping, the corresponding proton gradient, and the transfer of an e^-^ to molecular O_2_. Therefore, oxygen utilization predominately undergoes the acceptance of an electron during the synthesis of ATP. When oligomycin is injected into each well, a dramatic decrease in oxygen consumption rates is directly related to ATP synthesis. Mock cells had a more pronounced oxygen utilization for the purpose of ATP synthesis, measured as oxygen consumption rate (OCR), compared to infected cells (OCR (237±19 vs. 110±18 pmol/min/Cells, p-value<0.001), **Figure 5C**. Direct comparison between HEK (**Figure 3**) and Huh7 (**Figure 5**) cell data show similar trends, despite that specific values and data points are different due to the nature of the various cell lines used for the experiments.

### Mitochondrial Fusion Promoter, M1 Did Not Affect Viral Replication

**Figure 6:**
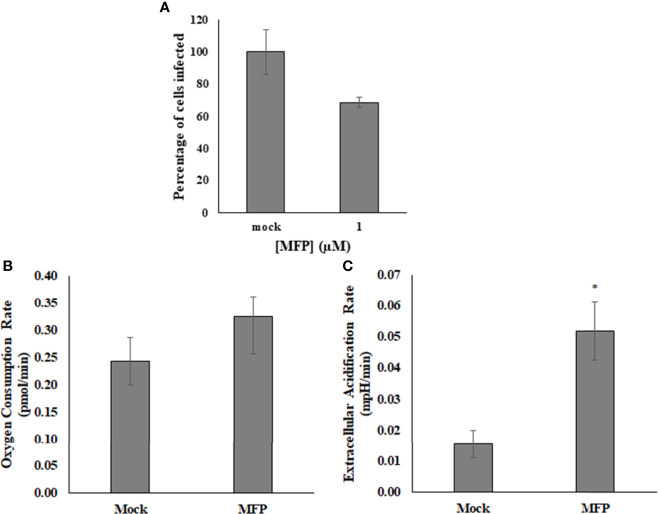
M1/MFP did not show any antiviral activity, but altered the host cell metabolism when used at a concentration of 100 µM. When used as a treatment for SINV (MOI=1), there was no significant alteration to viral output **(A)**. The OCR and ECAR readings were determined at a concentration of 100uM MFP in uninfected cells. There is no significant different in the oxygen consumption rate, p = 0.3 **(B)**, but the extracellular acidification rate is significantly different from the mock, untreated sample, p = 0.005 **(C)**. Mock= untreated.

M1 targets the mitofusin proteins and induces mitochondrial elongation *via* fusion and is referred to as the Mitochondrial Fusion Promoter (MFP) It has been shown to act selectively on cells with fragmented mitochondria, and has no effect on wild type or healthy cells (Diaz et al., 2010; Wang et al., 2010). The use of this compound was intended to determine whether SINV was affecting the mitochondrial morphology for replication. It was found that up to 100µM of M1/MFP treatment, there was no change in either HEK cell viability or in the viral output of any cell sample, based on detection of the viral reporter This compound did however cause an increase in OCR and ECAR in uninfected cells (**Figure 6**). This indicates that the TCA cycle or at least oxygen consumption is increased with treatment, but did not enhance or help viral replication.

## Discussion

The data indicates that Sindbis virus infection utilizes non-mitochondrial metabolism during infection, or at least reduces the dependence on mitochondrial metabolism. Normal cellular respiration relies heavily on mitochondrial respiration, including the TCA cycle and electron transport chain to generate the ATP and metabolites required to sustain cellular physiology. However, there are other pathways that can be used to generate ATP and be used to sustain cells during stressful conditions. Glycolysis is the main catabolic pathway that oxidizes glucose into pyruvate and generates 2ATP from each glucose molecule. Pyruvate can enter the TCA cycle as acetyl-CoA during aerobic respiration where it is further oxidized in several steps to generate ATP. In an anaerobic state, pyruvate is reduced into lactate regenerating NAD^+^for continuous use through glycolysis. The formation of lactate in the absence of oxygen is known as fermentation. However, when pyruvate is fermented into lactate in the presence of oxygen, this is notably referred to as the Warburg effect, a common characteristic found in cancer cells (Yang et al., 2014). Cancer cells maintain high rates of glucose uptake to support the demand for glucose-6-phosphate shunting through the pentose phosphate pathway for nucleotide synthesis that will further lead to cell division. This non-mitochondrial metabolic pathway can support cancer cells. Viral infections have been known to cause cancer, and some viruses show similar characteristics to cancer cells, with metabolic regulations that proceed to lactate fermentation in the presence of oxygen (Bäck and Lundkvist, 2013; Sanchez and Lagunoff, 2015; Mazzon et al., 2018). A Warburg-like effect is familiar to many +ssRNA viruses and has been linked to the robust in-take of glucose during infection (Strauss and Strauss, 1994; Findlay and Ulaeto, 2015; Fontaine et al., 2015). The Warburg-like effect is also believed to play a similar function in the synthesis of nucleotides for viral genomes. This metabolic phenotype and switch away from mitochondrial metabolism may benefit the virus by focusing resources to biomolecule anabolism instead of ATP production in the mitochondria.

SINV-infected cells had dysfunctional mitochondria shown by decreased oxygen consumption rates that were measured in real-time at 24 hours post infection. Mitochondrial dysfunction was confirmed by evaluating the basal and maximal respiratory rates during SINV-infection, both of which were significantly reduced compared to healthy, uninfected cells. These reduced oxygen consumption rates impacted the amount of respiration for ATP synthesis and indicate that SINV infection reduced mitochondrial metabolism. The most significant findings were that SINV infection depressed all aspects of mitochondrial respiration in HEK cells. Basal OCR levels measured before the injection of oligomycin were dramatically depressed in SINV infected vs. Mock Huh7 cells. After basal respiration was evaluated, oligomycin was injected to determine the amount of oxygen utilization related to ATP synthesis. SINV showed significant decreases in OCR, thus, at least some of the basal respiration was linked to ATP synthesis. The OCR readings for Mock cells dropped most dramatically, indicating high usage of oxygen for the production of ATP, after rotenone/antimycin (R/A) antibiotic treatment. Interestingly, SINV-infected cells showed only a subtle drop in OCR, indicating a low level of oxygen utilization for ATP production. Treatment of mitochondria with MFP increased oxygen consumption, but had no effect on increasing virus replication, complementing our hypothesis that virus replication is not directly correlated with mitochondrial ATP production.

With the increase in lactate accumulation and the reduction in mitochondrial metabolism, we can conclude that Sindbis Virus infection preferentially uses non-mitochondrial metabolism at 24 hour post infection of cultured cells.

## Data Availability Statement

The original contributions presented in the study are included in the article/**Supplementary Material**. Further inquiries can be directed to the corresponding author.

## Author Contributions

JC, JR, and JS conceived, designed, and orchestrated the work. AG and JS acquired funding and provided critical direction and oversight for the project. JC, JR, KY, and MS collected and analyzed the results. All authors read and approved the final manuscript. All authors contributed to the article and approved the submitted version.

## Funding

CBASE, Biology Department USAFA, Defense Threat Reduction Agency Service Academy Research Initiative (SARI).

## Author Disclaimer

The views expressed in this article, book, or presentation are those of the author and do not necessarily reflect the official policy or position of the United States Air Force Academy, the Air Force, the Department of Defense, or the U.S. Government.

## Conflict of Interest

The authors declare that the research was conducted in the absence of any commercial or financial relationships that could be construed as a potential conflict of interest.

## Publisher’s Note

All claims expressed in this article are solely those of the authors and do not necessarily represent those of their affiliated organizations, or those of the publisher, the editors and the reviewers. Any product that may be evaluated in this article, or claim that may be made by its manufacturer, is not guaranteed or endorsed by the publisher.

## Supplementary Material

Supplementary Figure 1 BHK cells were infected with dsGFPSINV at an increasing MOI (uninfected, 0.1, 1, and 10). At 24 hours post infection, the percentage of infected cells was determined based on fluorescent reporter **(A)**. The cells were also stained with resazurin for a viability assay and the results are shown **(B)**.

Supplementary Figure 2 Data profiles from . The Seahorse data obtained shows a change as the virus infection (MOI) increases. Full data set available upon request.
