# Supplementary figures and images for: Sindbis Virus Replication Reduces Dependence on Mitochondrial Metabolism During Infection
Source: Front Cell Infect Microbiol. 2022 Jun 16;12:859814. doi: 10.3389/fcimb.2022.859814 (PMC9245453; doi:10.3389/fcimb.2022.859814)

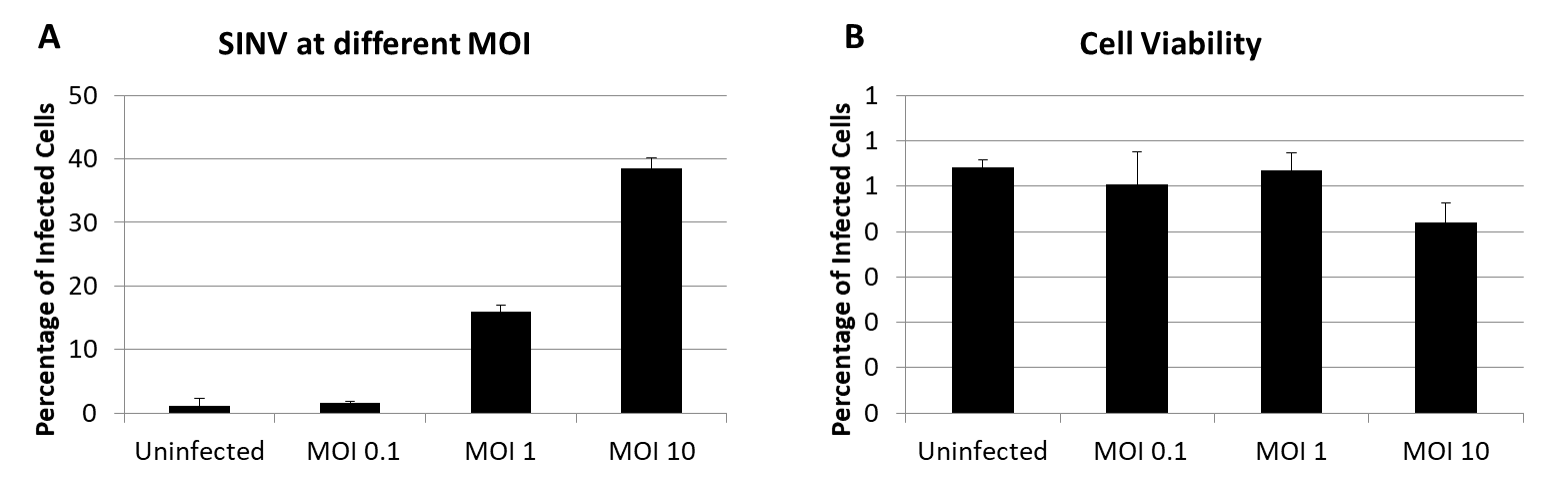

Supplement: Supplementary Figure 1 — BHK cells were infected with dsGFPSINV at an increasing MOI (uninfected, 0.1, 1, and 10). At 24 hours post infection, the percentage of infected cells was determined based on fluorescent reporter (A). The cells were also stained with resazurin for a viability assay and the results are shown (B). [file Image_1.tif]

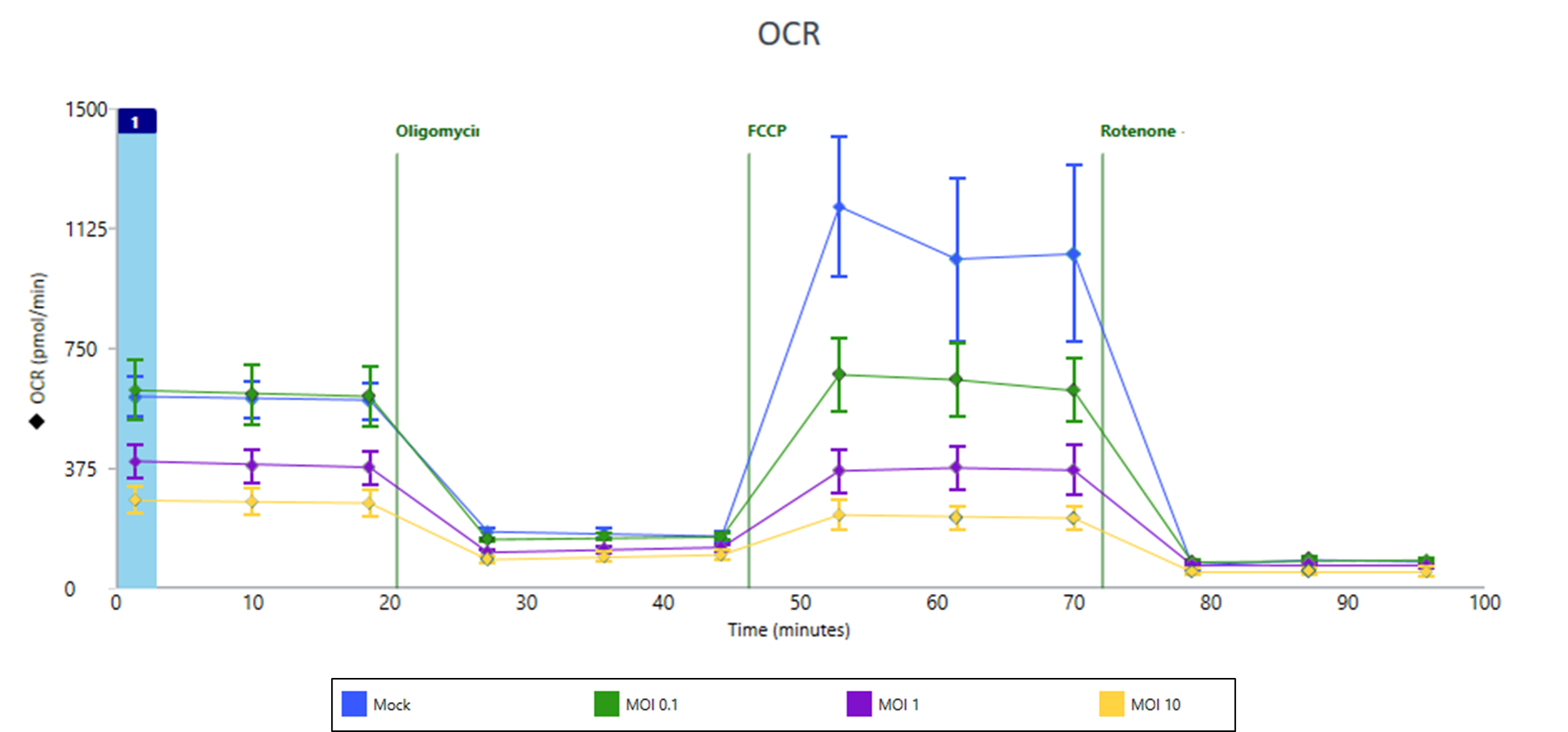

Supplement: Supplementary Figure 2 — Data profiles from . The Seahorse data obtained shows a change as the virus infection (MOI) increases. Full data set available upon request. [file Image_2.tif]
